# Factors associated with infection-related hospitalisations in severe mental illness: a retrospective cohort study

**DOI:** 10.1136/bmjment-2026-302636

**Published:** 2026-06-29

**Authors:** Amy Ronaldson, Sarah Markham, Alex Dregan, Temi Lampejo, Jayati Das-Munshi, Matthew Broadbent, Claire Henderson, Robert Stewart, Ioannis Bakolis

**Affiliations:** 1 Health Service and Population Research Department, Institute of Psychiatry, Psychology and Neuroscience (IoPPN), King’s College London, London, UK; 2 Department of Biostatistics & Health Informatics, IoPPN, King’s College London, London, UK; 3 Lived Experience Advisory Panel member, London, UK; 4 Department of Psychological Medicine, King’s College London, London, UK; 5 Infection Sciences, King’s College Hospital NHS Foundation Trust, London, UK; 6 South London and Maudsley NHS Foundation Trust, London, UK; 7 NIHR Maudsley Biomedical Research Centre (BRC), London, UK

**Keywords:** Schizophrenia, Bipolar and Related Disorders, Mental Health Services, Psychotic Disorders

## Abstract

**Background:**

People with severe mental illness (SMI) have a higher risk of infection-related hospitalisations than the general population, yet the reasons why remain poorly understood.

**Objective:**

We aimed to identify factors associated with infection-related hospitalisations and to explore how these factors cluster together within the patient population.

**Methods:**

We conducted a retrospective cohort study using linked electronic health records from a large secondary mental health service in South London. Individuals with an SMI diagnosis between 1 January 2007 and 31 December 2019 were included. Cox regression models examined associations between a wide range of sociodemographic, health-related, clinical and treatment and service-use factors and time to first infection-related hospitalisation. Significant factors were then used in a hierarchical cluster analysis to identify distinct patterns.

**Findings:**

In 19 995 individuals with SMI, several factors associated with infection-related hospitalisations emerged, with the strongest being number of general hospital admissions for ambulatory care sensitive conditions (HR=2.31, 95% CI 2.08 to 2.56), clozapine prescribing (HR=1.67, 95% CI 1.45 to 1.92) and severe problems with physical illness/disability (HR=1.36, 95% CI 1.31 to 1.42). Two distinct patterns emerged: one characterised by older age, dementia and poor physical health; the other by younger age with complex psychiatric needs and use of alcohol and other substances.

**Conclusions1:**

This study identified several factors associated with infection-related hospitalisations and highlighted how these factors tend to group together among people with SMI.

**Clinical implications:**

These findings indicate potential areas where tailored prevention and monitoring strategies may be warranted to help reduce the likelihood of infection-related hospitalisation in people with SMI alongside broader measures such as prioritisation for pneumococcal and influenza vaccination.

**WHAT IS ALREADY KNOWN ON THIS TOPIC**

- People with severe mental illness (SMI) experience substantially higher rates of infection-related morbidity, including increased hospitalisations for COVID-19, pneumonia and a wide range of other infections, even after accounting for age and medical comorbidity.

**WHAT THIS STUDY ADDS**

- This study adds to existing evidence by describing specific factors associated with infection-related hospitalisation in people with SMI using detailed linked mental health records. It also explores, for the first time, how these factors cluster into two broad patterns: one characterised by older age and physical illness; and another by younger age with more complex psychiatric and substance-use needs.

**HOW THIS STUDY MIGHT AFFECT RESEARCH, PRACTICE OR POLICY**

- These findings may help inform future work on how infection prevention and monitoring could be incorporated within existing physical health pathways for people with SMI, including approaches such as structured infection-risk assessment or vaccine review. They also point to potential opportunities for service-level exploration—for example, whether settings such as clozapine clinics could support proactive screening and vaccination—which could inform future research and service planning.

## Introduction

 People with severe mental illness (SMI) are at greater risk of poor physical health than the general population,^1^ leading to reduced life expectancy.^2^ Infectious disease is a significant contributor to excess mortality in people with SMI, who are more than two times as likely to die from any infection, and more than three times more likely to die from influenza and pneumonia,^3^ compared with the general population.

SMI is linked to worse infection-related outcomes besides mortality. During the COVID-19 pandemic, people with SMI were more likely to be hospitalised than those without SMI.^4^ This vulnerability extends to other respiratory infections like pneumonia, which occurs more often in people with SMI^5^ and leads to higher rates of pneumonia-related hospitalisation.^6^ More generally, patients with schizophrenia show elevated risk of hospitalisation for almost all types of hospital-registered infections.^7^ Broader investigations have found that people with depression and mental health service users are more likely to be hospitalised for infections, including vaccine-preventable ones.^8 9^

Few studies have explored why people with SMI are at such increased risk. Evidence suggests hospitalisation occurs at a younger age in SMI,^8^ and that medical comorbidity further amplifies this risk.^7^ However, these differences are not fully explained by differences in age, and associations survive adjustment for comorbid medical conditions.^6^ A large Danish registry study reported younger age, female gender, medical comorbidity and substance use contributed to higher likelihood of infection-related hospital contacts among people with schizophrenia.^10^

Using a retrospective cohort of individuals with SMI, we examined a wide range of factors potentially associated with infection-related hospitalisations, spanning sociodemographic characteristics, health-related factors, clinical features and treatment and service-use patterns. Our primary aim was to describe these associations, and we also explored how these factors clustered within the patient population.

## Methods

### Study design and participants

We created a retrospective cohort using electronic health records at South London and Maudsley National Health Service (NHS) Foundation Trust (SLaM). SLaM is one of Europe’s largest secondary mental healthcare providers, covering an urban catchment area of approximately 1.36 million people. SLaM established the Clinical Record Interactive Search (CRIS) system in 2008, which provides researchers with access to deidentified mental healthcare data.^11^ CRIS contains clinician-recorded information for all individuals in contact with SLaM since 1 January 2007 and is linked with Hospital Episode Statistics (HES),^12^ which hold details of admitted patient care, outpatient appointments and emergency department (ED) attendances at NHS hospitals in England. This study is reported in accordance with the Strengthening the Reporting of Observational Studies in Epidemiology (STROBE) guidelines.

Our study sample comprised all service users who received a primary diagnosis of SMI between 1 January 2007 and 31 December 2019. We ended follow-up in 2019 to avoid the confounding effects of the COVID-19 pandemic and the disrupted healthcare access experienced by people with SMI during that period. SMI was defined according to codes from the International Classification of Diseases, 10th Edition (ICD-10). Individuals with schizophrenia, schizotypal and delusional disorders (F2*), and bipolar disorder (F30, F31) were identified from prestructured diagnosis fields and supplemented by a natural language processing application developed with Generalised Architecture for Text Engineering. We included individuals who were ≥16 years at the time of first diagnosis. We excluded people who had received a diagnosis of dementia (F0*) before their SMI diagnosis to minimise diagnostic misclassification; however, diagnoses predating CRIS coverage may not be captured. Follow-up periods in the study were from date of first SMI diagnosis (study start date) to date of first infection-related hospitalisation, date of death or study end date (31 December 2019).

### Potential factors associated with infection-related hospitalisation

Potential factors were selected based on data availability, existing literature and input from the study’s Lived Experience Advisory Panel (LEAP). These factors spanned four domains: sociodemographic characteristics, health-related factors, clinical features and treatment and service-use patterns. For variables with possible multiple values over time (eg, smoking status, BMI), we used the most recently recorded value and treated it as a fixed variable. Because the dates of these entries were unavailable, they were conceptualised as relatively stable characteristics, and it is possible that some values were recorded after the study outcome. The timing of measurement for all variables is summarised in online supplemental table S1. Associations involving variables measured in the study window (ie, study start date to 31 December 2019) should be interpreted as associational rather than causal or predictive.

Sociodemographic characteristics included age at SMI diagnosis, gender, ethnicity, marital status at initial presentation and neighbourhood deprivation. Ethnicity was categorised into seven ethnic groups, dictated by data availability and sample size: white (White British, White Other), Black Caribbean, Black African, Black Other, South Asian (Bangladeshi, Indian, Pakistani), Mixed ethnicity and Other (eg, Chinese, Arab/Middle Eastern). Marital status was recorded as married/cohabiting, separated/divorced, widowed or single. Neighbourhood deprivation was measured using the Index of Multiple Deprivation (IMD) 2019 scores at the lower-layer super output area (LSOA) level based on the most recent address. IMD provides a metric of deprivation based on seven domains using specific weightings.^13^ IMD was presented as quintiles to facilitate comparison.

Health-related factors included smoking status and body mass index (BMI). Smoking status detailed whether an individual was a current smoker, past smoker or never smoked. The most recently recorded BMI (kg/m^2^) was categorised using established cut-offs (underweight <18.5, normal weight: 18.5 to <25, overweight: 25 to <30, obese: 30 to <40, severe obesity ≥40).

Clinical features included type of SMI diagnosis (F2* or F30, F31), mental health comorbidities, relevant Health of the Nation Outcome Scales (HoNOS) and emergency hospital admissions for ambulatory care-sensitive conditions (ACSCs). Mental health comorbidities were modelled as conditions recorded within the follow-up period based on ICD-10 diagnostic codes. HoNOS is a 12-item clinician-rated measure of health and social functioning.^14^ Three HoNOS items identified by the LEAP to be relevant to infection-related outcomes were included: problem drinking/drug-taking; physical illness/disability; and problems with activities of daily living. Each was scored from 0 (no problem) to 4 (severe problem). Using linkage with HES, we identified the number of emergency admissions for ACSCs during follow-up. ACSCs are conditions that should be managed outside of hospital (eg, asthma, diabetes), and emergency admissions for these conditions may indicate inadequate community or primary care support.^15^ Further details are provided in online supplemental table S2.

Treatment and service-use patterns comprised mental health service use and antipsychotic prescribing. Mental health service use included number of inpatient admissions and associated bed days, Home Treatment Team events, Community Mental Health Team events, emergency attendances and detention under the Mental Health Act. The total number of different antipsychotics (online supplemental table S3) prescribed during the study period were included as a measure of polypharmacy, potentially reflecting poor tolerability or inadequate response. Clozapine exposure was defined as ever having a clozapine prescription recorded during the study period, used as a marker of treatment-resistant schizophrenia.

### Infection-related hospitalisations

The main outcome was first infection-related hospitalisation in the study period. HES were used to identify infection-related hospitalisations. Because HES reports data in episodes, we defined an infection-related hospitalisation as any admission with an ICD-10 infection code (online supplemental table S4) as the primary diagnosis, indicating infection was the main reason for hospitalisation.

### Statistical analysis

Variables were summarised as medians and IQRs and proportions. Group differences were assessed using Mann-Whitney U and χ^2^ tests.

We first performed four domain-specific Cox proportional hazard regression models evaluating: (1) sociodemographic characteristics, (2) health-related factors, (3) clinical features and (4) treatment and service use patterns. Models for domains 2 to 4 were adjusted for age, gender, ethnicity and neighbourhood deprivation. Variables found to be statistically significant were subsequently entered into a final integrated model to identify factors most strongly associated with time to infection-related hospitalisation. Deaths occurring before an infection-related hospitalisation were treated as censoring events; the models, therefore, estimate cause-specific hazards. We computed HRs with 95% CIs. The timescale was calendar time (days) and the proportional hazards assumption was assessed using Schoenfeld residuals.^16^ Multicollinearity was assessed using variance inflation factors (VIFs).

Data were missing for several variables: ethnicity (7.5%), marital status (17.2%), IMD (7.0%), smoking status (32.2%), BMI (49.7%) and the HoNOS items (32.2 to 32.5%). Multiple imputation using chained equations with 10 imputations was performed to handle missing data (detail in Supplementary Methods). All Cox regression models were based on imputed data.

To examine how factors associated with infection-related hospitalisation grouped into patterns within the patient population, significant factors from the final model were entered into an agglomerative hierarchical cluster analysis using weighted-average linkage and Gower’s distance. The optimal number of clusters was determined using the Calinski-Harabasz and Duda-Hart indices. Differences between clusters were assessed using Mann-Whitney U and χ^2^ tests. Cluster analysis used complete cases because imputation can impose model-based structure and reduce natural variability.

All analyses were conducted using STATA V.18.0 (Stata Corp LLP, College Station, Texas).

### Sensitivity analyses

As the missing-at-random (MAR) assumption might not hold when using routinely collected data, we carried out complete-case analysis to confirm findings from imputed estimates.

Where Schoenfeld residuals indicated that proportional hazard assumptions had been violated, we fitted Poisson regression models with time-split person-time as a sensitivity analysis to account for differential follow-up and censoring without relying on the proportional hazards assumption. Follow-up was split into 1-year intervals and the log of person-time was included as an offset variable.

### Involvement of people with lived experience

This study was supported by a LEAP of four people with relevant lived experience. They met five times with the first author (AR) to plan and discuss the study. LEAP members contributed to shaping the research question, guiding the selection of variables and the analysis, interpreting results and drafting the final write-up.

## Results

### Sample selection and characteristics

The final study sample comprised 19 995 SLaM service users with SMI first recorded between 1 January 2007 and 31 December 2019, after excluding 53 service users who were diagnosed after the study end date (online supplemental figure S1). Sample characteristics are detailed in table 1.

**Table 1:** Sample characteristics by infection hospitalisation status (N=19 995)

	Overall (N=19 995)	Infection-related hospitalisation (N=2542, 12.7%)	No hospitalisation (N=17 453, 87.3%)	P value
	Median (IQR) or N (%)	Median (IQR) or N (%)	Median (IQR) or N (%)	
Sociodemographic factors				
Age at SMI diagnosis	37.7 (27.6 to 50.4)	52.6 (37.0 to 69.0)	36.3 (27.0 to 48.0)	<0.001
Female	9293 (46.5)	1297 (51.0)	7996 (45.8)	<0.001
Ethnicity				<0.001
White	9266 (50.1)	1523 (62.6)	7743 (48.2)	
Mixed	524 (2.8)	44 (1.8)	480 (3.0)	
South Asian	632 (3.4)	82 (3.4)	550 (3.4)	
Black Caribbean	1312 (7.1)	202 (8.3)	1110 (6.9)	
Black African	2315 (12.5)	211 (8.8)	2100 (13.1)	
Black Other	2158 (11.7)	190 (7.8)	1968 (12.3)	
Other	2280 (12.3)	175 (7.2)	2105 (13.1)	
Missing	*1508*	*111*	*1397*	
IMD quintile				0.117
1 (most deprived)	3733 (20.1)	518 (21.1)	3215 (19.9)	
2	3702 (19.9)	472 (19.3)	3230 (20.0)	
3	3733 (20.1)	481 (19.6)	3252 (20.1)	
4	3715 (20.0)	462 (18.8)	3253 (20.2)	
5	3706 (19.9)	518 (21.1)	3188 (19.7)	
Missing	*1406*	*91*	*1315*	
Marital status				<0.001
Married/cohabiting	2778 (16.8)	407 (17.7)	2371 (16.6)	
Separated/divorced	1583 (9.6)	295 (12.8)	1288 (9.0)	
Widowed	554 (3.3)	230 (10.0)	324 (2.3)	
Single	11 649 (70.3)	1365 (59.4)	10 284 (72.1)	
Missing	*3431*	*245*	*3186*	
Health-related factors				
Smoking status				0.007
Current smoker	10 183 (75.1)	1268 (72.1)	8915 (75.7)	
Past smoker	241 (1.8)	36 (2.0)	205 (1.7)	
Non-smoker	3140 (23.1)	456 (25.9)	2684 (22.7)	
Missing	*6431*	*782*	*5649*	
Body mass index				<0.001
Underweight	452 (4.5)	70 (5.7)	382 (4.3)	
Normal weight	3856 (38.4)	425 (34.4)	3431 (38.9)	
Overweight	2934 (29.2)	347 (28.1)	2587 (29.3)	
Obese	2375 (23.6)	310 (25.1)	2065 (23.4)	
Severe obesity	436 (4.3)	83 (6.7)	353 (4.0)	
Missing	*9942*	*1307*	*8635*	
Clinical features				
Index SMI diagnosis				0.179
Schizophrenia spectrum disorders	14 662 (73.3)	1836 (72.2)	12 826 (73.5)	
Bipolar disorder	5333 (26.7)	706 (27.8)	4627 (26.5)	
Mental health comorbidities				
F0*	761 (3.8)	367 (14.4)	394 (2.3)	<0.001
F1*	2300 (11.5)	314 (12.3)	1986 (11.4)	0.151
F3 (excl F30, F31)	2635 (13.2)	383 (15.1)	2252 (12.9)	0.003
F4*	1914 (9.6)	243 (9.6)	1671 (9.6)	0.981
F5*	346 (1.7)	33 (1.3)	313 (1.8)	0.074
F6*	1309 (6.5)	190 (7.5)	1119 (6.4)	0.043
F7*	535 (2.7)	115 (4.5)	420 (2.4)	<0.001
F8*	427 (2.1)	38 (1.5)	389 (2.2)	0.017
Emergency hospital admission for ACSC	1109 (5.5)	504 (19.8)	605 (3.5)	<0.001
HoNOS substance use	1 (1 to 2)	1 (1 to 1)	1 (1 to 2)	<0.001
Missing	*6500*	*731*	*5769*	
HoNOS physical illness	1 (1 to 2)	2 (1 to 4)	1 (1 to 2)	<0.001
Missing	*6442*	*723*	*5719*	
HoNOS activities of daily living	1 (1 to 2)	2 (1 to 3)	1 (1 to 2)	<0.001
Missing	*6460*	*728*	*5732*	
Treatment and service use patterns				
Number of psychiatric inpatient admissions	1 (0 to 3)	0 (0 to 3)	1 (0 to 3)	<0.001
Psychiatric inpatient bed days	10 (0 to 85)	0 (0 to 107)	13 (0 to 83)	0.003
Home treatment team events	0 (0 to 8)	0 (0 to 2)	0 (0 to 9)	<0.001
Community mental health team events	16 (3 to 63)	23 (4 to 79)	15 (2 to 62)	<0.001
Mental Health Act ever	7776 (38.9)	836 (32.9)	6940 (39.8)	<0.001
Emergency attendances	0 (0 to 2)	0 (0 to 2)	0 (0 to 2)	0.001
Clozapine prescribed	1506 (7.5)	262 (10.3)	1244 (7.1)	<0.001
Number of antipsychotic types prescribed				0.006
0	4552 (22.8)	534 (21.0)	4018 (23.0)	
1	6676 (33.4)	829 (32.6)	5847 (33.5)	
2	3645 (18.2)	454 (17.9)	3191 (18.3)	
3	2099 (10.5)	270 (10.6)	1829 (10.5)	
4+	3023 (15.1)	455 (17.9)	2568 (14.7)	
Mortality	2683 (13.4)	1116 (43.9)	1567 (9.0)	

F0*=Dementia, F1*=substance use disorders, F3*=mood disorders (excluding F30 and F31), F4*=neurotic, stress-related and somatoform disorders, F5*=behavioural syndromes associated with physiological disturbances and physical factors, F6*=disorders of adult personality and behaviour, F7*=intellectual disability, F8*= disorders of psychological development.

ACSC, Ambulatory care sensitive condition; HoNOS, Health of the Nation Outcome Scale; IMD, Index of Multiple Deprivation; SLaM, South London and Maudsley NHS Trust; SMI, severe mental illness.

The median age at diagnosis for the cohort was 37.7 years, and 46.5% were female. Half of the sample were of white ethnicity (50.1%), and most were single (70.3%). Most patients had an index diagnosis of schizophrenia spectrum disorders (73.3%). During follow-up, 12.7% (N=2542) had an infection-related hospitalisation. Among these admissions, respiratory infections were most common (39.3%), followed by urinary/renal infections (20.4%) and skin infections (15.8%) (online supplemental table S5). Among those who had an infection-related hospitalisation, 43.9% died during the follow-up period compared with 9.0% of those not hospitalised. Median follow-up was 5.7 years (IQR=2.4 to 9.3). Those with an infection-related hospitalisation had shorter follow-up (3.0 years, IQR=1.1 to 5.8) than those without (6.2 years, IQR=2.7 to 9.6).

### Factors associated with infection-related hospitalisations in people with SMI

Several factors were associated with infection-related hospitalisations across the four domain-specific Cox models (online supplemental table S6). Sociodemographic characteristics included age at diagnosis, ethnicity, IMD, and marital status. BMI was the only significant health-related factor. Associated clinical features associated were comorbid F0*, F1* and F7* diagnoses, emergency hospital admissions for ACSCs, and HoNOS items. Treatment and service use factors included clozapine prescribing and number of psychiatric inpatient admissions, Community Mental Health Team events, emergency attendances and detention under the Mental Health Act.

**Figure 1:**
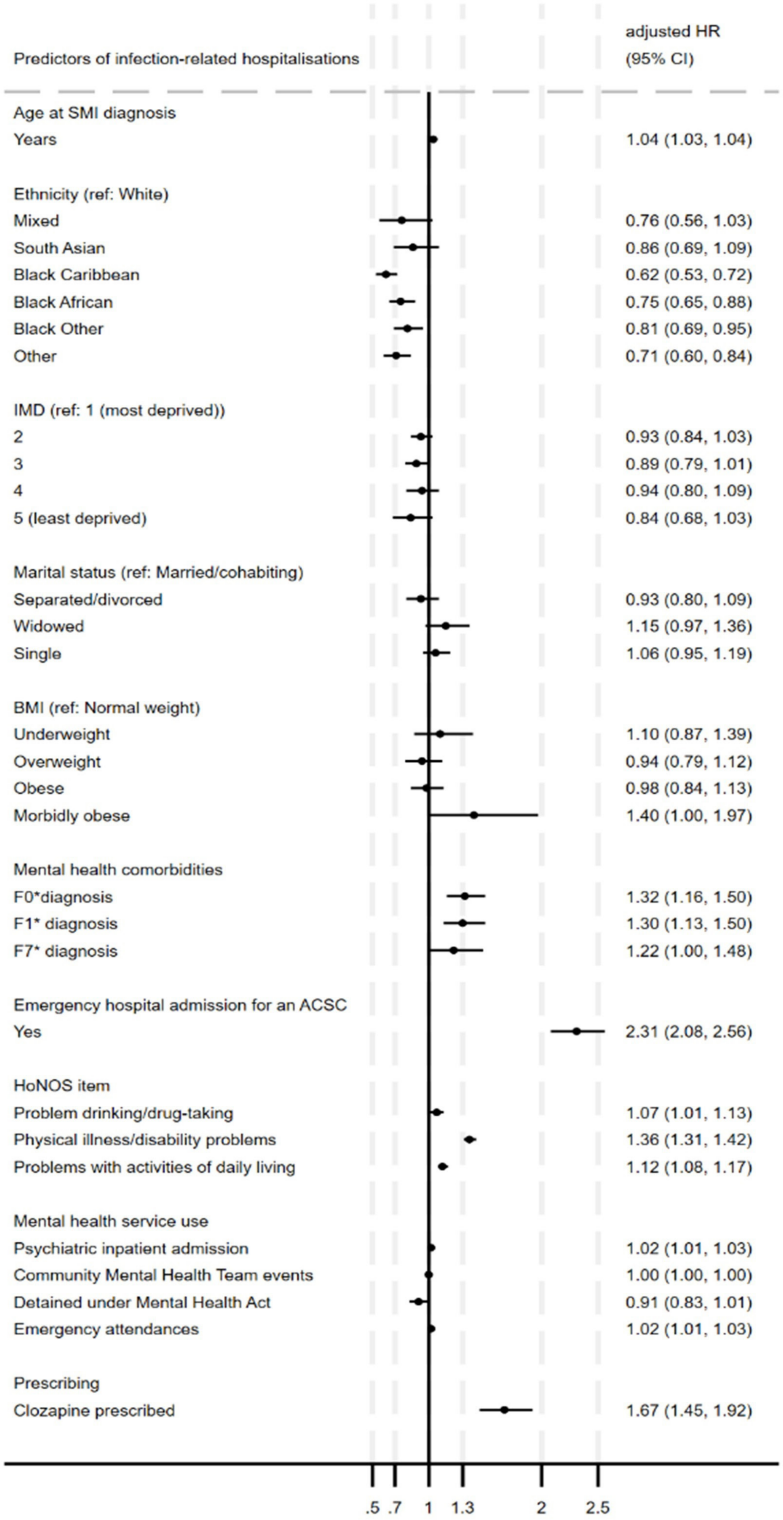
Factors associated with infection-related hospitalisations in people with SMI. Hazard ratios >1 denote increased risk of infection-related hospitalisation for that exposure. ACSC, Ambulatory care sensitive condition; BMI, body mass index; HoNOS, Health of the Nation Outcome Scale; IMD, Index of Multiple Deprivation; SLaM, South London and Maudsley NHS Trust; SMI, severe mental illness.

All significant factors from the domain-specific models were entered into a final integrated model presented in figure 1.

In the final model, older age at SMI diagnosis was associated with increased likelihood of infection-related hospitalisation (HR=1.04, 95% CI 1.03 to 1.04). Compared with white individuals, those from black or other ethnic groups had lower likelihood (eg, Black Caribbean HR=0.62, 95% CI 0.53 to 0.72). Significant clinical factors included receipt of a F0* (dementia) (HR=1.32, 95% CI 1.16 to 1.50) and a F1* (substance use disorder (SUD)) diagnosis (HR=1.30, 95% CI 1.13 to 1.50). Higher HoNOS scores for problem drinking/drug-taking (HR=1.07, 95% CI 1.01 to 1.13), physical illness/disability (HR=1.36, 95% CI 1.31 to 1.42) and problems with activities of daily living (HR=1.12, 95% CI 1.08 to 1.17) were also associated with infection-related hospitalisations. Emergency admissions for ACSCs showed a strong association (HR=2.31, 95% CI 2.08 to 2.56). Significant service use factors included number of inpatient admissions (HR=1.02, 95% CI 1.01 to 1.03) and emergency attendances (HR=1.02, 95% CI 1.01 to 1.03). Receipt of a clozapine prescription was associated with increased likelihood also (HR=1.67, 95% CI 1.45 to 1.92).

### Patient patterns: cluster analysis

Significant factors from the final integrated model were entered into a cluster analysis performed on N=13 045 (65.2%) with complete data for each variable. Individuals included in the cluster analysis differed from those excluded on several variables, reflecting the higher level of missingness among excluded participants (online supplemental table S7). Calinski-Harabasz and Duda-Hart indices supported a two-cluster solution. Differences between clusters are presented in table 2.

**Table 2:** Hierarchical cluster analysis of significant risk factors for infection-related hospitalisations (N=13 045)

	Cluster 1 (N=12 371)	Cluster 2 (N=674)	P value
	Median (IQR) or N(%)	M±SD or N(%)	
Age at diagnosis (M±SD)	39.7±15.8	67.3±16.4	<0.001
Ethnicity			
White	5654 (45.7)	374 (55.5)	<0.001
Mixed	371 (3.0)	14 (2.1)	
South Asian	420 (3.4)	19 (2.8)	
Black Caribbean	976 (7.9)	119 (17.7)	
Black African	1758 (14.2)	63 (9.3)	
Black Other	1695 (13.7)	39 (5.8)	
Other	1497 (12.1)	46 (6.8)	
Mental health comorbidities			
F0*	0 (0.0)	674 (100.0)	<0.001
F1*	1749 (14.1)	50 (7.4)	<0.001
Clozapine prescription	1076 (8.7)	26 (3.9)	<0.001
Inpatient admissions	2 (0 to 5)	1 (0 to 3)	<0.001
Emergency attendances	1 (0 to 3)	0 (0 to 1)	<0.001
Emergency hospital admission for ACSC	635 (5.1)	140 (20.8)	<0.001
HoNOS substance	1 (1 to 2)	1 (1 to 1)	<0.001
HoNOS physical	1 (1 to 2)	3 (2 to 4)	<0.001
HoNOS activities of daily living	1 (1 to 2)	3 (1 to 4)	<0.001
Infection hospitalisations	1444 (11.7)	336 (49.8)	<0.001
Sepsis	105 (0.8)	25 (3.7)	<0.001
Respiratory infection	570 (4.6)	121 (17.9)	<0.001
GI infection	133 (1.1)	26 (3.9)	<0.001
Renal/urinary infection	278 (2.2)	129 (19.1)	<0.001
CNS infection	8 (0.06)	2 (0.3)	0.034
Skin infection	238 (1.9)	24 (3.6)	0.003
HIV/Hepatitis	9 (0.07)	0 (0.0)	0.484

Hierarchical cluster analysis was performed using the significant risk factors for infection-related hospitalisation that emerged from the final integrated model. The cluster analysis was performed on patients with complete data on all relevant variables.

ACSC, ambulatory care sensitive condition; CNS, central nervous system; F0*, dementia; F1*, substance use disorders; GI, gastrointestinal; HoNOS, Health of the Nation Outcome Scales; M, mean.

Cluster 1 (N=12 371) comprised younger service users, more often from black or other ethnic groups, with higher rates of SUD and clozapine prescribing, and more psychiatric inpatient admissions and emergency attendances. Cluster 2 (N=674) comprised older, predominantly white service users with poorer physical health, greater functional impairment, more emergency admissions for ACSCs and universal dementia. Although both clusters experienced infection-related hospitalisations, rates were markedly higher in cluster 2 across most infection types.

### Sensitivity analysis

Results from complete case analyses are presented in online supplemental table S8a,b. Complete case estimates led to some changes to the domain-specific models including the removal of IMD and detentions under the Mental Health Act as significant factors, and the addition of smoking status. The final integrated model (N=7585) revealed results similar to the imputed model with additional significant factors including severe obesity and the removal of F0 and F1 diagnoses.

Schoenfeld residuals (online supplemental table S9–S13) indicated violations of the proportional hazards assumption in models examining clinical features (online supplemental table S11), treatment and service use patterns (online supplemental table S12) and in the final integrated model (online supplemental table S13). The Poisson regression sensitivity analysis using time-split person-time produced incidence rate ratios (online supplemental table S14) almost identical to those produced using Cox regression, indicating that the main findings were robust to violations of the proportional hazards assumption.

VIFs for each model are provided in online supplemental table S15–S19. Mean VIFs were low across all models and no individual variable had a VIF of ≥10, a commonly used threshold for identifying problematic multicollinearity.^17^ Therefore, multicollinearity was not deemed to be a concern.

## Discussion

This study identified several sociodemographic, clinical and treatment-related factors associated with infection-related hospitalisation in people with SMI. Older age at SMI diagnosis was associated with higher rates of hospitalisations, while individuals from black and other ethnic groups had lower rates than white individuals. Clinical factors associated with hospitalisation included dementia, SUD, emergency admissions for ACSCs and higher HoNOS scores for problem drinking/drug use, physical illness and difficulties with daily living. Treatment and service-use characteristics associated with higher rates included more psychiatric inpatient admissions, more emergency psychiatric attendances and clozapine prescribing. Cluster analysis revealed two distinct patterns: an older group characterised by poor physical health and dementia and a younger group characterised by SUD, clozapine prescribing and high psychiatric service use. These profiles suggest differing underlying pathologies and pathways to infection-related hospitalisation.

Infection-related hospitalisations can occur for a wide range of reasons, including true underlying infection incidence, infection severity or clinical concerns unrelated to infection (eg, self-neglect prompting admission where an incidental infection is recorded). Hospital admission may also increase the likelihood of an infection being coded as a diagnosis on discharge. The factors identified in this study could associate or interact with any of these pathways in different ways. However, the data available did not allow us to distinguish between these underlying mechanisms.

### Sociodemographic factors

We found that older age at SMI diagnosis was associated with higher rates of infection-related hospitalisation. Although this contrasts with a Danish study reporting greater risk in younger individuals,^10^ it aligns with evidence from COVID-19 showing increased vulnerability in older adults.^4^ Later-life SMI diagnoses may reflect atypical or complex clinical presentations, contributing to poorer infection outcomes.

In contrast to previous studies,^18^ we found that individuals from black and other ethnic backgrounds had lower rates of infection-related hospitalisation. This finding should be interpreted with caution, as differences in service-use patterns, pathways into care or unmeasured physical health and social factors might also contribute, and residual confounding cannot be ruled out.

### Clinical features

Dementia was strongly associated with higher rates of infection-related hospitalisation, consistent with its known link to poor infection outcomes and the challenges detecting and managing infections in this group.^19 20^ This association may be particularly relevant among people with SMI. In line with findings from Denmark,^10^ comorbid SUD and more severe scores on the HoNOS problem drink/drug-taking item were also associated with higher hospitalisation rates. HoNOS indicators of poor physical health and disability were similarly associated. Substance misuse, poor physical health and long-term conditions have been linked to impaired immune function and increased infection-related complications,^21 22^ and more frequent contact with healthcare services may increase exposure to healthcare-associated infections.^23^

Emergency ACSC hospital admissions may be linked with infection-related hospitalisations through the shared mechanisms outlined above, but they might also signal inadequate community or primary care support,^15^ while also reflecting broader illness burden in some cases. People with SMI experience well-documented barriers to timely and effective healthcare,^24^ which may lead to delayed help-seeking, more complex clinical presentations, and infections that might be managed in the community progressing to require admission.

### Treatment and service use patterns

More frequent psychiatric inpatient admissions and emergency psychiatric events might associate with infection-related admissions as part of an overall picture of increased health service use among some of this SMI cohort. However, these aspects of psychiatric service use might indicate more severe, clinically complex or poorly managed SMI. Severity of psychiatric symptoms is known to be associated with poor infection outcomes.^25^ Moreover, psychiatric inpatient units face unique infection-control challenges, which may increase infection risk in these environments.^26^

Clozapine prescription was associated with a higher risk of infection-related hospitalisation, consistent with evidence linking it to increased incidence and severity of infections, particularly respiratory infections such as pneumonia and COVID-19.^27 28^ Potential mechanisms might include clozapine-associated inflammation, agranulocytosis and sedation and hypersalivation, which may have consequences for aspiration pneumonia in particular. Some of this association might reflect confounding by indication, as clozapine is prescribed for more severe or treatment-resistant illness, and clinicians may admit clozapine-treated patients more readily when infection is suspected. Because clozapine prescribing was coded as an ever/never variable, the observed association likely reflects a composite of treatment resistance, illness severity, survival to treatment initiation and patterns of service contact, in addition to any drug-specific effect.

### Patient patterns

Data-driven clustering summarised how key factors co-occurred in this cohort and revealed two distinct patient patterns. Individuals included in the cluster analysis differed from those excluded due to higher missingness in several variables. The smaller cluster comprised older individuals with SMI, poor physical health and dementia and high rates of infection-related hospitalisation—patterns consistent with infection risk in the general population.^19 29^ The other comprised younger individuals with higher SUD, clozapine prescribing and intensive service contact, with comparatively lower infection-related hospitalisation rates. This suggests infection-related hospitalisations for younger people with SMI may relate more to factors such as substance use, treatment complexity and crisis-driven care than physical health or frailty.

### Implications for practice

Current NHS policies aim to address physical health inequalities experienced by people with SMI, including annual physical health checks and tailored outreach and health promotion. Our findings may help inform thinking about how infection prevention could be considered within these existing pathways. For example, annual physical health checks could potentially include more structured discussions about infection risk and vaccine uptake, particularly for individuals with repeated emergency ACSC admissions, those prescribed clozapine, and those with comorbid SUD. As smoking is often part of SUD, ensuring access to smoking cessation support in primary and secondary care may also be relevant, given that respiratory infections were the most common cause of infection-related hospitalisation in our cohort.

People with SMI are not currently prioritised for routinely offered vaccines such as influenza or pneumococcal vaccination in the UK. Given the patterns observed in our study, these findings may help inform future considerations of how vaccination pathways are delivered and accessed by people with SMI. These findings may also point to areas for future service-level exploration. For example, clozapine clinics have been suggested as potential settings for proactive physical health activity, and our results raise the question of whether infection-related discussions, respiratory assessment and vaccine reviews could be incorporated into such contacts. For older adults with SMI, dementia, and poor physical health, our findings highlight the importance of considering how mental health and geriatric services might work together to support infection-related care needs. For younger people with SMI and SUD, integrated approaches that bring together substance use and physical health support may warrant further exploration.

### Strengths and limitations

We used comprehensive electronic health records linked with national hospital admission data, which allowed infection-related hospitalisations to be assessed for up to 13 years using primary diagnosis codes to ensure high specificity. As well as examining associations across a wide range of sociodemographic, clinical and treatment-related factors, we also applied data-driven techniques to further refine our understanding of how these factors group together in.

This study has several limitations. Findings are generalisable only to individuals with SMI who accessed secondary mental health services within a given time period and will have missed those with limited or no service contact. Results are most applicable to South London and similar urban settings and may not extend to regions with different healthcare structures. The observational study design precludes causal inference and although we adjusted for a wide range of factors, unmeasured confounding remains possible. For example, we could not adjust for the presence of specific physical long-term conditions, many of which are managed entirely in primary care and, therefore, not comprehensively captured in the hospital admission data available to us. Moreover, we could not account for vaccine status. As infection-related hospitalisations were largely respiratory in nature (39%), the absence of influenza and pneumococcal vaccination data prevented us from determining whether increased infection-related hospitalisations partially reflected lower vaccine uptake or reduced effectiveness in this population. As the MAR assumption may not fully hold for several covariates, some residual uncertainty due to missing data remains. Furthermore, due to the nature of the data linkage, we could not adjust for infection hospitalisations pre-SMI diagnosis. As our outcome was restricted to primary infection diagnoses, the number of events within specific infection categories was too small to allow meaningful subgroup analyses, and infection-related admissions, therefore, remain a heterogeneous group. Some factors (eg, smoking status, BMI) were treated as fixed variables using their most recently recorded value, which may have introduced misclassification if these characteristics changed over time. Only individuals with complete data were included in the cluster analysis, which may introduce selection bias, although the patterns observed are coherent and clinically plausible.

## Conclusion

People with SMI face a substantial burden of infection-related hospitalisation. This study identified several sociodemographic, clinical and treatment-related factors associated with these hospitalisations. The data-driven patient groupings that emerged suggest that vulnerability to infection-related hospitalisation in SMI is not uniform but may arise through different constellations of factors. These findings highlight potential areas for considering tailored approaches to infection-related prevention and monitoring within this population, including management of physical health, support for groups with more complex needs and consideration of how vaccination and other preventative activities are delivered and accessed.

### Lived experience commentary

From a lived experience perspective, this study is of high relevance to people with SMI. A key message from this work is that people living with SMI do not experience the same level of vulnerability to serious infections. Patterns in the data suggest that risk varies across groups, with differences observed by age, physical health, substance use and how mental healthcare is delivered, highlighting the value of evidence-informed personalised approaches to care. The findings further underline the close ties between physical and mental health. People with SMI who also have long-term physical conditions, disability or dementia were found to be much more likely to be hospitalised for infections. For people with lived experience, this reinforces that concerns about physical health symptoms are valid, deserve prompt attention and should not be neglected or ignored. Higher rates of hospital admissions and potentially preventable hospitalisations suggest that some people may not be receiving the help they need sufficiently early. From a lived-experience perspective, this reflects well-known barriers such as difficulty accessing general practitionaers (GPs), not being taken seriously, or struggling to manage treatment when unwell. The results support calls for better, more proactive physical healthcare and follow-up in the community. For people prescribed clozapine, the findings appear to be of significant concern and might indicate the need for better monitoring and support to access effective treatment. The two, quite distinct patient patterns provide evidence for targeted personalised prevention and support.

The key message for people with lived experience is that poor infection outcomes are not inevitable. Many of the factors found to be linked to hospitalisation reflect system-level issues; how care is organised, monitored and accessed. The study supports advocacy for better physical healthcare, respectful treatment, early intervention and prevention (including vaccination), alongside mental healthcare. It may be of preventative value for future research to examine associations between primary care contact and/or vaccination uptake and hospitalisation for infectious disease in people with SMI.

## Supplementary material

10.1136/bmjment-2026-302636 online supplemental file 1

## Data Availability

Data may be obtained from a third party and are not publicly available.
