# Supplementary material for: Factors associated with infection-related hospitalisations in severe mental illness: a retrospective cohort study
Source: BMJ Ment Health. 2026 Jun 29;29(1):e302636. doi: 10.1136/bmjment-2026-302636 (PMC13331047; doi:10.1136/bmjment-2026-302636)
Supplement: online supplemental file 1 [file bmjment-29-1-s001.docx]

**Risk factors for infection-related hospitalisations in severe mental illness: A retrospective cohort study**

**Supplementary Material**

**Supplementary Methods**

| **Table S1.** Timing of measurement for all potential factors associated with infection-related hospitalisation | |
| --- | --- |
| Age | At cohort entry |
| Gender | At cohort entry |
| Ethnicity | Ever recorded |
| Marital status | At cohort entry |
| Neighbourhood deprivation | Based on address closest to study end date |
| Smoking status | Most recent value prior to study end date |
| Body mass index | Most recent value prior to study end date |
| SMI diagnosis | At cohort entry |
| Mental health comorbidities | During study period |
| HoNOS items | Closest to cohort entry |
| Emergency admissions for ACSCs | During study period |
| Mental health service use | During study period |
| Number of different antipsychotics prescribed | During study period |
| Clozapine prescribing | During study period |
| Cohort entry=date of first SMI diagnosis  Study end date= 31 December 2019  Study period=Date of first SMI diagnosis to 31 December 2019 (regardless of exiting the cohort due outcome) | |

*Defining ACSCs*

In the current study ACSCs were defined according to NHS criteria which specifies 19 conditions divided into acute, chronic, and vaccine-preventable^1^. We included non-infectious conditions only to generate a measure of emergency hospital admissions for ACSCs (13/19 conditions). A list of included and excluded conditions and their associated ICD-10 codes are listed below. Emergency hospital admissions for ACSCs were extracted from Hospital Episode Statistics (HES) data linked with the SLaM CRIS dataset. HES reports in episodes (i.e. a period of care under a consultant) and there can be multiple episodes recorded in one hospital stay. Each episode can have multiple recorded diagnoses (ICD-10 codes) which were used to identify ACSCs. In the current study, an ACSC admission was defined as an admission where an ACSC condition was recorded in the first episode of the admission (SPELBGN=1, 2). The admission method also had to be considered emergency (ADMIMETH: 21, 22, 23, 24, 28).

| **Table S2.** Ambulatory Care Sensitive Conditions (ACSCs) | | |
| --- | --- | --- |
| **ACSC** | **ICD-10 codes** | **Included** |
| Angina | I20 I24.0 I24.8 I24.9 | Included |
| Asthma | J45 J46 | Included |
| Cellulitis | L03 L04 L08.0 L08.8 L08.9 L88 L98.0 | Excluded – infection |
| Congestive heart failure | I11.0 I50 J81 | Included |
| Convulsions, epilepsy | G40 G41 R56 O15 | Included |
| COPD | J20 J41 J42 J43 J47 | Included – J20 excluded as infection |
| Dehydration and gastroenteritis | E86 K52.2 K52.8 K52.9 | Included |
| Dental conditions | A69.0 K02 K03 K04 K05 K06 K08 K09.8 K09.9 K12 K13 | Included – A69.0 excluded as infection |
| Diabetes | E10 E11 E12 E13 E14 | Included |
| ENT infections | H66 H67 J02 J03 J06 J31.2 | Excluded – infection |
| Gangrene | R02 | Included |
| Hypertension | I10 I11.9 | Included |
| Flu and pneumonia | J10 J11 J13 J14 J15.3 J15.4 J15.7 J15.9 J16.8 J18.1 J18 | Excluded – infection |
| Iron-deficiency anaemia | D50.1 D50.8 D50.9 | Included |
| Nutritional deficiency | E40 E41 E42 E43 E55.0 E64.3 | Included |
| Other vaccine-preventable diseases | A35 A36 A37 A80 B05 B06 B16.1 B16.9 B18.0 B18.1 B26 G00.0 M01.4 | Excluded – infection |
| Pelvic inflammatory disease | N70 N73 N74 | Excluded – infection |
| Bleeding ulcer | K25.0–K25.2 K25.4–K25.6 K26.0–K26.2 K26.4–K26.6 K27.0–K27.2 K27.4–K27.6 K28.0–28.2 K284–K286 | Included |
| Pyelonephritis | N10 N11 N12 N13.6 | Excluded - infection |

*Antipsychotic medications*

| **Table S3.** List of antipsychotic medications |
| --- |
| Amisulpride, Aripiprazole, Asenapine, Benperidol, Cariprazine, Chlorpromazine, Clozapine, Droperidol, Flupentixol, Fluphenazine, Haloperidol, Levomepromazine, Lurasidone, Melperone, Olanzapine, Paliperidone, Penfluridol, Pericyazine, Perphenazine, Pimozide, Piportil, Pipotiazine, Promazine, Quetiapine, Risperidone, Sulpiride, Thioridazine, Trifluoperazine, Ziprasidone, Zuclopenthixol |

*Defining infection hospitalisations*

| **Table S4.** ICD-10 codes used to define infection hospitalisations (primary diagnosis only) | |
| --- | --- |
| **Infection Category** | **ICD-10 code** |
| Any infection | A00-B99, G00, G01, G02, G03, H62.0, H62.1, H67.0, H67.1, J00, J01, J02, J03, J04, J05, J06, J09, J10, J11, J12, J13, J14, J15, J16, J17, J18, J20, J21, J22, L00, L01, L02, L03, L04, L05, L08, M00, M01, N10, N11, N13.6, N30, N39.0, N45, N70, N71, N72, N73.0, N73.1, N73.2, N73.3, N73.4, N73.5, N74.0, N74.1, N74.2, N74.3, N74.4, N76.4, N77.0, N77.1, O23, O26.4, O85, O86, O98, R57.2, R65.0, R65.1 |
| Sepsis | A40-A41, R57.2 |
| Respiratory infections |  |
| *Influenza* | J09-J11 |
| *Pneumonia* | J12-J18 |
| *Other respiratory* | J00-J06, J20-J22 |
| Gastrointestinal infections | A00-A09 |
| Renal/urinary infections | N10, N11, N13.6, N30, N39.0, N45, N70, N71, N72, N73.0, N73.1, N73.2, N73.3, N73.4, N73.5, N74.0, N74.1, N74.2, N74.3, N74.4, N76.4, N77.0, N77.1 |
| CNS infections | A17, A32.1, A39.0, A81, A83-A89, B00.3, B00.4, B01.0, B01.1, B02.0, B02.1, B02.2, B05.0, B05.1, B06.0, B26.1, B26.2, B58.2, G00-G03 |
| Skin infections | A46, B00-B09, L00-L08 |
| HIV or hepatitis infections | B15-B19, B20-B24 |

***Multiple imputations by chained equations***

63.8% (N=12,750) of the sample had missing data on at least one of the proposed risk factors in the study: ethnicity (N=1508, 7.5%), marital status (N=3431, 17.2%), IMD (N=1406, 7.0%), smoking status (N=6431, 32.2%), BMI (N=9942, 49.7%), HoNOS items (N=6442 (32.2%) to 6500 (32.5%)). Multiple imputation by chained equations (MICE) was performed for these missing data within Stata 18.0 (“mi impute chained”) for all patients in the cohort. For imputations, ethnicity, marital status, IMD decile, smoking status, and BMI category were specified as categorical/multinomial variables using “mlogit”, and the HoNOS items were specified as continuous using “regress”. Variables with no missing values were included in MICE: Age at diagnosis, gender, index SMI diagnosis, mental health comorbidities, mental health service use variables, antipsychotic prescribing, clozapine prescribing, hospital admissions for ACSCs, and infection hospitalisations. We imputed 10 datasets using a random seed of 1234.

*References*

1 Purdy S, Griffin T, Salisbury C, Sharp D. Ambulatory care sensitive conditions: terminology and disease coding need to be more specific to aid policy makers and clinicians. *Public Health* 2009; **123**: 169–73.

**Supplementary Results**

| **Table S5.** Infection hospitalisations (N=2542, 12.7%) | |
| --- | --- |
| *Infection type* | *Number of hospitalisations*, N(%)* |
| Sepsis | 195 (7.7) |
| Respiratory | 1000 (39.3) |
| Gastrointestinal | 231 (9.1) |
| Renal/urinary | 519 (20.4) |
| CNS | 16 (0.6) |
| Skin | 401 (15.8) |
| HIV/Hepatitis | 10 (0.4) |
| *First hospitalisations | |

| **Table S6. Domain-specific associations between risk factors and time to first infection-related hospitalisations** | | | | | | | |
| --- | --- | --- | --- | --- | --- | --- | --- |
| **Sociodemographic characteristics** | | **Health-related factors†** | | **Clinical features†** | | **Treatment and service use patterns†** | |
|  | *HR (95% CI)* |  | *HR (95% CI)* |  | *HR (95% CI)* |  | *HR (95% CI)* |
| Age at diagnosis | 1.05 (1.04 to 1.05)** | Smoking status |  | Index SMI diagnosis |  | Number of psychiatric inpatient admissions | 1.02 (1.01 to 1.03)* |
| Female | 0.94 (0.86 to 1.02) | *Current smoker* | Reference | *Schizophrenia spectrum disorders* | Reference | Psychiatric inpatient bed days | 1.00 (1.00 to 1.00) |
| Ethnicity |  | *Past smoker* | 0.99 (0.67 to 1.43) | *Bipolar disorder* | 1.03 (0.94 to 1.13) | HTT events | 1.00 (1.00 to 1.00) |
| *White* | Reference | *Non-smoker* | 0.89 (0.80 to 1.00) | Mental health comorbidities |  | CMHT events | 1.00 (0.99 to 1.00)* |
| *Mixed* | 0.81 (0.60 to 1.11) | Body mass index |  | *F0* | 1.30 (1.15 to 1.48)** | MHA ever | 0.88 (0.79 to 0.98)* |
| *South Asian* | 0.82 (0.65 to 1.03) | *Normal weight* | Reference | *F1* | 1.21 (1.05 to 1.40)* | Emergency events | 1.02 (1.02 to 1.03)** |
| *Black Caribbean* | 0.64 (0.55 to 0.75)** | *Underweight* | 1.31 (1.00 to 1.71)* | *F3 (excl F30, F31)* | 1.04 (0.93 to 1.17) | Clozapine prescribed | 1.65 (1.42 to 1.91)** |
| *Black African* | 0.71 (0.61 to 0.82)** | *Overweight* | 0.91 (0.78 to 1.06) | *F4* | 1.08 (0.94 to 1.24) | Number of antipsychotic types prescribed |  |
| *Black Other* | 0.82 (0.70 to 0.96)* | *Obese* | 1.01 (0.88 to 1.17) | *F5* | 0.87 (0.61 to 1.23) | *0* | Reference |
| *Other* | 0.66 (0.56 to 0.77)** | *Severe obesity* | 1.72 (1.22 to 2.43)* | *F6* | 1.15 (0.98 to 1.35) | *1* | 0.99 (0.88 to 1.10) |
| IMD quintile |  |  |  | *F7* | 1.29 (1.05 to 1.59)* | *2* | 0.96 (0.84 to 1.09) |
| *1 (most deprived)* | Reference |  |  | *F8* | 0.83 (0.59 to 1.18) | *3* | 1.00 (0.85 to 1.17) |
| *2* | 0.97 (0.86 to 1.10) |  |  | Number of emergency hospital admission for ACSCs | 2.30 (2.07 to 2.55)** | *4+* | 1.12 (0.95 to 1.32) |
| *3* | 1.00 (0.89 to 1.14) |  |  | HoNOS problem drinking/drug-taking | 1.07 (1.01 to 1.14)* |  |  |
| *4* | 0.98 (0.86 to 1.11) |  |  | HoNOS physical illness/disability | 1.36 (1.30 to 1.41)** |  |  |
| *5 (least deprived)* | 1.15 (1.01 to 1.30)* |  |  | HoNOS ADL | 1.14 (1.09 to 1.19)** |  |  |
| Marital status |  |  |  |  |  |  |  |
| *Married/cohabiting* | Reference |  |  |  |  |  |  |
| *Separated/divorced* | 1.07 (0.92 to 1.25) |  |  |  |  |  |  |
| *Widowed* | 1.34 (1.12 to 1.59)* |  |  |  |  |  |  |
| *Single* | 1.24 (1.10 to 1.39)** |  |  |  |  |  |  |
| *p<0.05; **p<0.01  † Models adjusted for age at diagnosis, gender, ethnicity, and IMD  ACSC=ambulatory care sensitive condition; ADL=Activities of Daily Living; CI=confidence interval; HoNOS=Health of the Nations Outcome Scale; HR=hazard ratio; IMD=Index of Multiple Deprivation; SMI=severe mental illness | | | | | | | |

| **Table S7.** Sample characteristics for those included in the cluster analysis and those exclude from the cluster analysis | | | |
| --- | --- | --- | --- |
|  | **Cluster analysis**  **(N=13,045, 65.2%))** | **Excluded**  **(N=6950, 34.8%)** |  |
|  | *Median (IQR) or N (%)* | *Median (IQR) or N (%)* | *P value* |
| ***Sociodemographic factors*** |  |  |  |
| Age at SMI diagnosis | 37.8 (27.5 to 50.9) | 37.5 (27.9 to 49.7) | 0.030 |
| Female | 6236 (47.8) | 3057 (44.0) | <0.001 |
| Ethnicity |  |  | <0.001 |
| *White* | 6028 (46.2) | 3238 (59.5) |  |
| *Mixed* | 385 (2.9) | 139 (2.5) |  |
| *South Asian* | 439 (3.4) | 193 (3.5) |  |
| *Black Caribbean* | 1095 (8.4) | 217 (4.0) |  |
| *Black African* | 1821 (14.0) | 494 (9.1) |  |
| *Black Other* | 1734 (13.3) | 424 (7.8) |  |
| *Other* | 1543 (11.8) | 737 (13.5) |  |
| *Missing* | *0* | *1508* |  |
| IMD quintile |  |  | <0.001 |
| *1 (most deprived)* | 2011 (16.3) | 1722 (27.7) |  |
| *2* | 2424 (19.6) | 1278 (20.5) |  |
| *3* | 2626 (21.2) | 1107 (17.8) |  |
| *4* | 2747 (22.2) | 968 (15.6) |  |
| *5* | 2559 (20.7) | 1147 (18.4) |  |
| *Missing* | *678* | *728* |  |
| Marital status |  |  | <0.001 |
| *Married/cohabiting* | 2059 (17.0) | 719 (16.1) |  |
| *Separated/divorced* | 1237 (10.2) | 346 (7.8) |  |
| *Widowed* | 481 (4.0) | 73 (1.6) |  |
| *Single* | 8333 (68.8) | 3316 (74.4) |  |
| *Missing* | *935* | *2496* |  |
|  |  |  |  |
| ***Health-related factors*** |  |  |  |
| Smoking status |  |  | <0.001 |
| *Current smoker* | 7639 (72.6) | 2544 (83.7) |  |
| *Past smoker* | 202 (1.9) | 39 (1.3) |  |
| *Non-smoker* | 2684 (25.5) | 456 (15.0) |  |
| *Missing* | *2520* | *3911* |  |
| Body mass index |  |  | <0.001 |
| *Underweight* | 351 (4.1) | 101 (6.7) |  |
| *Normal weight* | 3219 (37.7) | 637 (42.3) |  |
| *Overweight* | 2531 (29.6) | 403 (26.7) |  |
| *Obese* | 2073 (24.3) | 302 (20.0) |  |
| *Severe obesity* | 372 (4.3) | 64 (4.2) |  |
| *Missing* | *4499* | *5443* |  |
|  |  |  |  |
| ***Clinical features*** |  |  |  |
| Index SMI diagnosis |  |  | <0.001 |
| *Schizophrenia spectrum disorders* | 9856 (75.5) | 4806 (69.1) |  |
| *Bipolar disorder* | 3189 (24.5) | 2144 (30.9) |  |
| Mental health comorbidities |  |  |  |
| *F0** | 674 (5.2) | 87 (1.2) | <0.001 |
| *F1** | 1799 (13.8) | 501 (7.2) | <0.001 |
| *F3 (excl F30, F31)* | 2149 (16.5) | 486 (7.0) | <0.001 |
| *F4** | 1481 (11.3) | 433 (6.2) | <0.001 |
| *F5** | 280 (2.1) | 66 (1.0) | <0.001 |
| *F6** | 1040 (8.0) | 269 (3.9) | <0.001 |
| *F7** | 242 (1.9) | 293 (4.2) | <0.001 |
| *F8** | 231 (1.8) | 196 (2.8) | <0.001 |
| Emergency hospital admission for ACSC | 775 (5.9) | 334 (4.8) | 0.001 |
| HoNOS substance use | 1 (1 to 1) | 1 (1 to 2) | 0.082 |
| *Missing* | *0* | *6500* |  |
| HoNOS physical illness | 1 (1 to 2) | 1 (1 to 2) | 0.001 |
| *Missing* | *0* | *6442* |  |
| HoNOS Activities of Daily Living | 1 (1 to 2) | 1 (1 to 2) | 0.032 |
| *Missing* | *0* | *6460* |  |
|  |  |  |  |
| ***Treatment and service use patterns*** |  |  |  |
| Number of psychiatric inpatient admissions | 2 (0 to 4) | 0 ( 0 to 0) | <0.001 |
| Psychiatric inpatient bed days | 42 (0 to 34) | 0 (0 to 0) | <0.001 |
| Home Treatment Team events | 1 (0 to 17) | 0 (0 to 0) | <0.001 |
| Community Mental Health Team events | 38 (11 to 99) | 2 (1 to 8) | <0.001 |
| Mental Health Act ever | 6492 (50.0) | 1284 (18.5) | <0.001 |
| Emergency attendances | 1 (0 to 3) | 0 (0 to 1) | <0.001 |
| Clozapine prescribed | 1102 (8.4) | 404 (5.8) | <0.001 |
| Number of antipsychotic types prescribed |  |  | <0.001 |
| *0* | 1335 (10.2) | 3217 (46.3) |  |
| *1* | 4285 (32.8) | 2391 (34.4) |  |
| *2* | 2911 (22.3) | 734 (10.6) |  |
| *3* | 1817 (13.9) | 282 (4.1) |  |
| *4+* | 2697 (20.7) | 326 (4.7) |  |
| Mortality | 1888 (14.5) | 795 (11.4) | <0.001 |
| Infection-related hospitalisation | 1780 (13.6) | 762 (11.0) | <0.001 |
| ACSC: Ambulatory care sensitive condition, HoNOS: Health of the Nation Outcome Scale, IMD: Index of Multiple Deprivation, SLaM: South London and Maudsley NHS Trust, SMI: severe mental illness  F0*=Dementia, F1*=substance use disorders, F3*=mood disorders (excluding F30 and F31), F4*=neurotic, stress-related and somatoform disorders, F5*=behavioural syndromes associated with physiological disturbances and physical factors, F6*=disorders of adult personality and behaviour, F7*=intellectual disability, F8*= disorders of psychological development | | | |

| **Table S8a. Complete case analyses (domain-specific)** | | | | | | | |
| --- | --- | --- | --- | --- | --- | --- | --- |
| **Sociodemographic characteristics**  **(N=15,462)** | | **Health-related factors†**  **(N=8597)** | | **Clinical features†**  **(N=12,367)** | | **Treatment and service use patterns†**  **(N=17,294)** | |
|  | *HR (95% CI)* |  | *HR (95% CI)* |  | *HR (95% CI)* |  | *HR (95% CI)* |
| Age at diagnosis | 1.05 (1.05 to 1.05)** | Smoking status |  | Index SMI diagnosis |  | Number of psychiatric inpatient admissions | 1.02 (1.01 to 1.03)* |
| Female | 0.94 (0.86 to 1.03) | *Current smoker* | Reference | *Schizophrenia spectrum disorders* | Reference | Psychiatric inpatient bed days | 1.00 (1.00 to 1.00) |
| Ethnicity |  | *Past smoker* | 0.98 (0.66 to 1.44) | *Bipolar disorder* | 1.02 (0.91 to 1.14) | HTT events | 1.00 (1.00 to 1.00) |
| *White* | Reference | *Non-smoker* | 0.84 (0.73 to 0.96)* | Mental health comorbidities |  | CMHT events | 1.00 (1.00 to 1.00) |
| *Mixed* | 0.82 (0.60 to 1.12) | BMI |  | *F0* | 1.32 (1.15 to 1.51)** | MHA ever | 0.90 (0.81 to 1.00) |
| *South Asian* | 0.80 (0.63 to 1.02) | *Normal weight* | Reference | *F1* | 1.28 (1.09 to 1.51)** | Emergency events | 1.02 (1.02 to 1.03)** |
| *Black Caribbean* | 0.65 (0.55 to 0.76)** | *Underweight* | 1.32 (1.00 to 1.73)* | *F3 (excl F30, F31)* | 1.13 (0.99 to 1.28) | Clozapine prescribed | 1.61 (1.38 to 1.87)** |
| *Black African* | 0.74 (0.64 to 0.87)** | *Overweight* | 0.94 (0.81 to 1.10) | *F4* | 1.10 (0.94 to 1.28) | Number of antipsychotic types prescribed |  |
| *Black Other* | 0.88 (0.75 to 1.03) | *Obese* | 1.07 (0.92 to 1.25) | *F5* | 0.89 (0.60 to 1.32) | *0* | Reference |
| *Other* | 0.67 (0.57 to 0.79)** | *Severe obesity* | 1.88 (1.46 to 2.42)** | *F6* | 1.13 (0.94 to 1.35) | *1* | 1.00 (0.89 to 1.13) |
| IMD quintile |  |  |  | *F7* | 1.41 (1.07 to 1.87)* | *2* | 0.96 (0.84 to 1.10) |
| *1 (most deprived)* | Reference |  |  | *F8* | 0.71 (0.43 to 1.17) | *3* | 0.99 (0.84 to 1.17) |
| *2* | 0.95 (0.83 to 1.09) |  |  | General hospital admission for ACSCs | 2.14 (1.89 to 2.42)** | *4+* | 1.11 (0.94 to 1.32) |
| *3* | 1.03 (0.90 to 1.17) |  |  | HoNOS problem drinking/drug-taking | 1.09 (1.02 to 1.16)* |  |  |
| *4* | 0.97 (0.85 to 1.11) |  |  | HoNOS physical illness/disability | 1.34 (1.29 to 1.40)** |  |  |
| *5* | 1.13 (0.99 to 1.29) |  |  | HoNOS ADL | 1.13 (1.08 to 1.18)** |  |  |
| Marital status |  |  |  |  |  |  |  |
| *Married/cohabiting* | Reference |  |  |  |  |  |  |
| *Separated/divorced* | 1.05 (0.90 to 1.23) |  |  |  |  |  |  |
| *Widowed* | 1.31 (1.10 to 1.56)* |  |  |  |  |  |  |
| *Single* | 1.24 (1.11 to 1.40)** |  |  |  |  |  |  |
| *p<0.05; **p<0.01  † Models adjusted for age at diagnosis, gender, ethnicity, and IMD  ACSC=ambulatory care sensitive condition; ADL=Activities of Daily Living; BMI=Body mass index; CI=confidence interval; CMHT=Community Mental Health Team; HoNOS=Health of the Nations Outcome Scale; HR=hazard ratio; HTT=Home Treatment Team; IMD=Index of Multiple Deprivation; MHA=Mental Health Act; SMI=severe mental illness | | | | | | | |

| **Table S8b. Complete case analyses (final integrated model) (N=7585)** | |
| --- | --- |
|  | HR (95% CI) |
| Age at diagnosis | 1.02 (1.01 to 1.03)* |
| Ethnicity |  |
| *White* | Reference |
| *Mixed* | 0.79 (0.53 to 1.20) |
| *South Asian* | 0.80 (0.54 to 1.17) |
| *Black Caribbean* | 0.66 (0.54 to 0.81)** |
| *Black African* | 0.69 (0.55 to 0.86)** |
| *Black Other* | 0.79 (0.63 to 0.99)* |
| *Other* | 0.75 (0.58 to 0.97)* |
| IMD quintile |  |
| *1 (most deprived)* | Reference |
| *2* | 0.95 (0.77 to 1.17) |
| *3* | 1.07 (0.87 to 1.30) |
| *4* | 0.99 (0.81 to 1.22) |
| *5* | 1.12 (0.92 to 1.38) |
| Marital status |  |
| *Married/cohabiting* | Reference |
| *Separated/divorced* | 0.99 (0.78 to 1.24) |
| *Widowed* | 1.22 (0.94 to 1.58) |
| *Single* | 1.08 (0.89 to 1.30) |
| BMI |  |
| *Normal weight* | Reference |
| *Underweight* | 0.95 (0.71 to 1.26) |
| *Overweight* | 0.93 (0.79 to 1.08) |
| *Obese* | 0.97 (0.82 to 1.14) |
| *Severe obesity* | 1.41 (1.07 to 1.85)* |
| Mental health comorbidities |  |
| F0 | 1.08 (0.89 to 1.31) |
| F1 | 1.16 (0.95 to 1.41) |
| F7 | 1.27 (0.92 to 1.77) |
| General hospital admission for ACSCs | 2.31 (1.97 to 2.70)** |
| HoNOS problem drinking/drug-taking | 1.12 (1.04 to 1.20)* |
| HoNOS physical illness/disability | 1.33 (1.26 to 1.41)** |
| HoNOS ADL | 1.09 (1.03 to 1.16)* |
| Number of psychiatric inpatient admissions | 1.02 (1.01 to 1.03)* |
| CMHT events | 1.00 (1.00 to 1.00)* |
| MHA ever | 1.02 (0.89 to 1.18) |
| Emergency events | 1.02 (1.01 to 1.02)** |
| Clozapine prescribed | 1.56 (1.30 to 1.88)** |
| *p<0.05; **p<0.01  ACSC=ambulatory care sensitive condition; ADL=Activities of Daily Living; BMI=Body mass index; CI=confidence interval; HoNOS=Health of the Nations Outcome Scale; HR=hazard ratio; IMD=Index of Multiple Deprivation | |

**Proportional hazard assumption: Schoenfeld Residuals**

| **Table S9.** Sociodemographic characteristics | | | |
| --- | --- | --- | --- |
|  | *chi2* | *p value* | *Interpretation* |
| Age at diagnosis | 0.01 | 0.931 | PH assumption holds |
| Female | 0.15 | 0.700 | PH assumption holds |
| Ethnicity |  |  |  |
| *White* | Ref |  |  |
| *Mixed* | 0.77 | 0.381 | PH assumption holds |
| *South Asian* | 0.19 | 0.664 | PH assumption holds |
| *Black Caribbean* | 0.59 | 0.442 | PH assumption holds |
| *Black African* | 1.42 | 0.233 | PH assumption holds |
| *Black Other* | 0.01 | 0.923 | PH assumption holds |
| *Other* | 2.03 | 0.154 | PH assumption holds |
| IMD quintile |  |  |  |
| *1 (most deprived)* | Ref |  |  |
| *2* | 2.32 | 0.128 | PH assumption holds |
| *3* | 2.57 | 0.109 | PH assumption holds |
| *4* | 0.22 | 0.643 | PH assumption holds |
| *5* | 3.69 | 0.055 | Potential violation |
| Marital status |  |  |  |
| *Married/cohabiting* | Ref |  |  |
| *Separated/divorced* | 0.15 | 0.696 | PH assumption holds |
| *Widowed* | 0.45 | 0.501 | PH assumption holds |
| *Single* | 0.07 | 0.792 | PH assumption holds |
| **Global test** | **10.81** | **0.766** | **PH assumption holds** |

| **Table S10.** Health-related factors | | | |
| --- | --- | --- | --- |
|  | *chi2* | *p value* | *Interpretation* |
| Smoking status |  |  |  |
| *Current smoker* | Ref | Ref |  |
| *Past smoker* | 0.64 | 0.422 | PH assumption holds |
| *Non-smoker* | 2.69 | 0.101 | PH assumption holds |
| BMI category |  |  |  |
| *Normal weight* | Ref | Ref |  |
| *Underweight* | 0.00 | 0.980 | PH assumption holds |
| *Overweight* | 0.07 | 0.785 | PH assumption holds |
| *Obesity* | 0.08 | 0.784 | PH assumption holds |
| *Severe obesity* | 0.04 | 0.839 | PH assumption holds |
| Age at diagnosis | 0.55 | 0.456 | PH assumption holds |
| Female | 1.56 | 0.212 | PH assumption holds |
| Ethnicity |  |  |  |
| *White* | Ref |  |  |
| *Mixed* | 0.11 | 0.741 | PH assumption holds |
| *South Asian* | 1.11 | 0.292 | PH assumption holds |
| *Black Caribbean* | 0.06 | 0.809 | PH assumption holds |
| *Black African* | 0.85 | 0.356 | PH assumption holds |
| *Black Other* | 0.10 | 0.753 | PH assumption holds |
| *Other* | 0.55 | 0.457 | PH assumption holds |
| IMD decile |  |  |  |
| *1 (most deprived)* | Ref |  |  |
| *2* | 1.40 | 0.237 | PH assumption holds |
| *3* | 3.05 | 0.081 | PH assumption holds |
| *4* | 0.14 | 0.707 | PH assumption holds |
| *5* | 5.42 | 0.020 | Potential violation |
| **Global test** | **15.23** | **0.646** | **PH assumption holds** |

| **Table S11.** Clinical features | | | |
| --- | --- | --- | --- |
|  | *chi2* | *p value* | *Interpretation* |
| SMI diagnosis |  |  |  |
| *Schizophrenia* | Reference |  |  |
| *Bipolar disorder* | 0.36 | 0.549 | PH assumption holds |
| Mental health comorbidities |  |  |  |
| *F0* | 0.75 | 0.386 | PH assumption holds |
| *F1* | 2.65 | 0.104 | PH assumption holds |
| *F3 (excl. F30, F31)* | 0.00 | 0.989 | PH assumption holds |
| *F4* | 0.07 | 0.788 | PH assumption holds |
| *F5* | 0.03 | 0.872 | PH assumption holds |
| *F6* | 3.01 | 0.082 | PH assumption holds |
| *F7* | 0.60 | 0.437 | PH assumption holds |
| *F8* | 0.38 | 0.536 | PH assumption holds |
| ACSC admissions | 14.55 | <0.001 | Potential violation |
| HoNOS substance use | 1.51 | 0.219 | PH assumption holds |
| HoNOS physical illness | 13.53 | <0.001 | Potential violation |
| HoNOS daily living | 0.44 | 0.508 | PH assumption holds |
| Age at diagnosis | 0.08 | 0.771 | PH assumption holds |
| Female | 0.32 | 0.570 | PH assumption holds |
| Ethnicity |  |  |  |
| *White* | Ref |  |  |
| *Mixed* | 0.00 | 0.966 | PH assumption holds |
| *South Asian* | 1.73 | 0.188 | PH assumption holds |
| *Black Caribbean* | 0.84 | 0.359 | PH assumption holds |
| *Black African* | 4.40 | 0.036 | Potential violation |
| *Black Other* | 0.62 | 0.430 | PH assumption holds |
| *Other* | 3.52 | 0.061 | PH assumption holds |
| IMD quintile |  |  |  |
| *1 (most deprived)* | Ref |  |  |
| *2* | 1.68 | 0.195 | PH assumption holds |
| *3* | 3.10 | 0.078 | PH assumption holds |
| *4* | 0.27 | 0.604 | PH assumption holds |
| *5* | 3.99 | 0.046 | Potential violation |
| **Global test** | **47.81** | **0.004** | **Potential violation** |

| **Table S12.** Treatment and service use patterns | | | |
| --- | --- | --- | --- |
|  | *chi2* | *p value* | *Interpretation* |
| Mental health service use |  |  |  |
| *Number of psychiatric inpatient admissions* | 0.18 | 0.669 | PH assumption holds |
| *Psychiatric inpatient bed days* | 0.01 | 0.927 | PH assumption holds |
| *HTT events* | 0.61 | 0.437 | PH assumption holds |
| *CMHT events* | 12.76 | <0.001 | Potential violation |
| *MHA ever* | 0.01 | 0.943 | PH assumption holds |
| *Emergency events* | 8.46 | 0.004 | Potential violation |
| Clozapine prescribed | 12.21 | <0.001 | Potential violation |
| Number of antipsychotic types prescribed |  |  |  |
| *0* | Reference |  |  |
| *1* | 1.32 | 0.250 | PH assumption holds |
| *2* | 1.90 | 0.168 | PH assumption holds |
| *3* | 5.23 | 0.022 | Potential violation |
| *4+* | 5.50 | 0.020 | Potential violation |
| Age at diagnosis | 0.01 | 0.915 | PH assumption holds |
| Female | 0.01 | 0.936 | PH assumption holds |
| Ethnicity |  |  |  |
| *White* | Ref |  |  |
| *Mixed* | 1.43 | 0.232 | PH assumption holds |
| *South Asian* | 0.48 | 0.487 | PH assumption holds |
| *Black Caribbean* | 0.52 | 0.472 | PH assumption holds |
| *Black African* | 1.57 | 0.211 | PH assumption holds |
| *Black Other* | 0.46 | 0.497 | PH assumption holds |
| *Other* | 2.08 | 0.149 | PH assumption holds |
| IMD quintile |  |  |  |
| *1 (most deprived)* | Ref |  |  |
| *2* | 0.95 | 0.330 | PH assumption holds |
| *3* | 0.86 | 0.355 | PH assumption holds |
| *4* | 0.02 | 0.881 | PH assumption holds |
| *5* | 0.71 | 0.399 | PH assumption holds |
| **Global test** | **51.70** | **<0.001** | **Potential violation** |

| **Table S13.** Final integrated model | | | |
| --- | --- | --- | --- |
|  | *chi2* | *p value* | *Interpretation* |
| Age at diagnosis | 0.01 | 0.937 | PH assumption holds |
| Ethnicity |  |  |  |
| *White* | Reference |  |  |
| *Mixed* | 0.06 | 0.803 | PH assumption holds |
| *South Asian* | 0.83 | 0.362 | PH assumption holds |
| *Black Caribbean* | 0.33 | 0.567 | PH assumption holds |
| *Black African* | 1.37 | 0.241 | PH assumption holds |
| *Black Other* | 0.30 | 0.584 | PH assumption holds |
| *Other* | 1.33 | 0.250 | PH assumption holds |
| IMD decile |  |  |  |
| *1 (most deprived)* | Reference |  |  |
| *2* | 0.86 | 0.353 | PH assumption holds |
| *3* | 2.12 | 0.145 | PH assumption holds |
| *4* | 0.97 | 0.325 | PH assumption holds |
| *5* | 2.01 | 0.156 | PH assumption holds |
| Marital status |  |  |  |
| *Married/cohabiting* | Reference |  |  |
| *Separated/divorced* | 0.25 | 0.615 | PH assumption holds |
| *Widowed* | 0.02 | 0.891 | PH assumption holds |
| *Single* | 0.18 | 0.672 | PH assumption holds |
| BMI category |  |  |  |
| *Normal weight* | Reference |  |  |
| *Underweight* | 0.07 | 0.798 | PH assumption holds |
| *Overweight* | 0.04 | 0.840 | PH assumption holds |
| *Obesity* | 0.06 | 0.800 | PH assumption holds |
| *Severe obesity* | 0.02 | 0.896 | PH assumption holds |
| Mental health comorbidities |  |  |  |
| *F0* | 1.13 | 0.289 | PH assumption holds |
| *F1* | 9.99 | 0.002 | Potential violation |
| *F7* | 2.61 | 0.106 | PH assumption holds |
| ACSC admissions | 6.36 | 0.012 | Potential violation |
| HoNOS substance use | 3.33 | 0.068 | PH assumption holds |
| HoNOS physical illness | 6.25 | 0.012 | Potential violation |
| HoNOS daily living | 0.93 | 0.334 | PH assumption holds |
| Clozapine prescription | 7.56 | 0.006 | Potential violation |
| Mental health serviced utilisation |  |  |  |
| *Number of acute episodes* | 0.00 | 0.993 | PH assumption holds |
| *Emergency events* | 8.76 | 0.003 | Potential violation |
| *MHA ever* | 0.06 | 0.803 | PH assumption holds |
| *CMHT events* | 11.00 | <0.001 | Potential violation |
| **Global test** | **53.55** | **0.005** | **Potential violation** |

**Sensitivity analysis: Poisson regression model with time‑split person‑time**

| **Table S14.** Associations between proposed risk factors and infection hospitalisation (Poisson regression models with time-split person-time) | | | |
| --- | --- | --- | --- |
|  | Clinical features | Treatment and service use patterns | Integrated model |
|  | *IRR (95% CI)* | *IRR (95% CI)* | *IRR (95% CI)* |
| Age at diagnosis | 1.04 (1.03 to 1.04)** | 1.05 (1.05 to 1.06)** | 1.04 (1.03 to 1.04)** |
| Female | 0.94 (0.86 to 1.02) | 0.94 (0.87 to 1.02) |  |
| Ethnicity |  |  |  |
| *White* | Reference | Reference | Reference |
| *Mixed* | 0.79 (0.58 to 1.08) | 0.82 (0.60 to 1.11) | 0.76 (0.56 to 1.03) |
| *South Asian* | 0.84 (0.67 to 1.05) | 0.81 (0.65 to 1.02) | 0.86 (0.69 to 1.09) |
| *Black Caribbean* | 0.61 (0.53 to 0.72)** | 0.62 (0.53 to 0.72)** | 0.61 (0.53 to 0.71)** |
| *Black African* | 0.75 (0.65 to 0.88)** | 0.69 (0.59 to 0.80)** | 0.76 (0.65 to 0.88)** |
| *Black Other* | 0.83 (0.71 to 0.98)* | 0.83 (0.71 to 0.97)* | 0.81 (0.69 to 0.95)* |
| *Other* | 0.70 (0.60 to 0.83)** | 0.67 (0.57 to 0.79)** | 0.71 (0.60 to 0.83)** |
| IMD quintile |  |  |  |
| *1 (most deprived)* | Reference | Reference | Reference |
| *2* | 0.93 (0.82 to 1.06) | 0.98 (0.87 to 1.12) | 0.93 (0.84 to 1.02) |
| *3* | 1.00 (0.88 to 1.13) | 1.02 (0.90 to 1.16) | 0.89 (0.79 to 1.00) |
| *4* | 0.97 (0.85 to 1.10) | 0.99 (0.87 to 1.13) | 0.93 (0.80 to 1.09) |
| *5* | 1.08 (0.95 to 1.22) | 1.18 (1.04 to 1.34)* | 0.84 (0.68 to 1.03) |
| Marital status |  |  |  |
| *Married/cohabiting* |  |  | Reference |
| *Separated/divorced* |  |  | 0.93 (0.79 to 1.08) |
| *Widowed* |  |  | 1.15 (0.97 to 1.37) |
| *Single* |  |  | 1.07 (0.95 to 1.20) |
| BMI |  |  |  |
| *Normal weight* |  |  | Reference |
| *Underweight* |  |  | 1.10 (0.87 to 1.39) |
| *Overweight* |  |  | 0.94 (0.79 to 1.12) |
| *Obese* |  |  | 0.97 (0.84 to 1.13) |
| *Severe obesity* |  |  | 1.40 (0.99 to 1.97) |
| SMI diagnosis |  |  |  |
| *Schizophrenia* | Reference |  |  |
| *Bipolar disorder* | 1.03 (0.94 to 1.13) |  |  |
| Mental health comorbidities |  |  |  |
| *F0* | 1.30 (1.14 to 1.47)** |  | 1.32 (1.16 to 1.50)** |
| *F1* | 1.21 (1.04 to 1.40)* |  | 1.31 (1.14 to 1.51)** |
| *F3 (excl. F30, F31)* | 1.04 (0.93 to 1.17) |  |  |
| *F4* | 1.09 (0.95 to 1.25) |  |  |
| *F5* | 0.86 (0.61 to 1.23) |  |  |
| *F6* | 1.14 (0.97 to 1.34) |  |  |
| *F7* | 1.28 (1.04 to 1.58)* |  | 1.21 (0.99 to 1.47) |
| *F8* | 0.83 (0.59 to 1.18) |  |  |
| ACSC Hospital Admissions | 2.30 (2.07 to 2.55)** |  | 2.31 (2.08 to 2.56)** |
| HoNOS substance | 1.07 (1.01 to 1.14)* |  | 1.07 (1.01 to 1.14)* |
| HoNOS physical | 1.36 (1.31 to 1.42)** |  | 1.37 (1.31 to 1.42)** |
| HoNOS ADL | 1.14 (1.09 to 1.19)** |  | 1.12 (1.08 to 1.17)** |
| Clozapine prescription |  | 1.67 (1.43 to 1.94)** | 1.68 (1.46 to 1.93)** |
| Number of different antipsychotic prescriptions |  |  |  |
| *0* |  | Reference |  |
| *1* |  | 0.98 (0.88 to 1.10) |  |
| *2* |  | 0.94 (0.83 to 1.08) |  |
| *3* |  | 0.98 (0.84 to 1.15) |  |
| *4+* |  | 1.10 (0.93 to 1.30) |  |
| *Number of acute episodes* |  | 1.02 (1.01 to 1.03)* | 1.02 (1.00 to 1.03)* |
| *Acute bed days* |  | 1.00 (0.99 to 1.00) |  |
| *HTT events* |  | 1.00 (1.00 to 1.00) |  |
| *CMHT events* |  | 0.999 (0.998 to 0.999)* | 1.00 (1.00 to 1.00)** |
| *MHA ever* |  | 0.89 (0.80 to 0.99) | 0.92 (0.83 to 1.01) |
| *Emergency events* |  | 1.02 (1.02 to 1.03)** | 1.02 (1.01 to 1.03)** |
| *p<0.05, **p<0.001  ACSC=ambulatory care sensitive condition; ADL=Activities of Daily Living; CI=confidence interval; CMHT=Community Mental Health Team; HoNOS=Health of the Nations Outcome Scale; HTT=Home Treatment Team; IMD=Index of Multiple Deprivation; IRR=incident rate ratio; MHA=Mental Health Act;; SMI=severe mental illness | | | |

**Multicollinearity: Variance Inflation Factors**

VIFS: To assess multicollinearity among the independent variables, we calculated the Variance Inflation Factors (VIFs). VIFs quantify how much the variance of a regression coefficient is inflated due to multicollinearity with other predictors. A VIF value of 1 indicates no multicollinearity, values between 1 and 5 suggest low to moderate multicollinearity, and values above 5 indicate moderate to high multicollinearity. VIF values exceeding 10 were considered indicative of severe multicollinearity. If any individual VIF exceeded 10, or the mean VIF for the model suggested substantial overall multicollinearity, we planned to take corrective action. The VIFs were calculated using STATA after running the regression model on the unimputed dataset.

| **Table S15.** Sociodemographic characteristics | | |
| --- | --- | --- |
|  | *VIF* | *Interpretation* |
| Age at diagnosis | 6.40 | Moderate to high |
| Female | 7.03 | Moderate to high |
| Ethnicity |  |  |
| *White* | Ref |  |
| *Mixed* | 1.07 | Low |
| *South Asian* | 1.07 | Low |
| *Black Caribbean* | 1.17 | Low |
| *Black African* | 1.30 | Low |
| *Black Other* | 1.30 | Low |
| *Other* | 1.24 | Low |
| IMD quintile |  |  |
| *1 (most deprived)* | Ref |  |
| *2* | 1.93 | Low |
| *3* | 1.99 | Low |
| *4* | 2.01 | Low |
| *5* | 1.99 | Low |
| Marital status |  |  |
| *Married/cohabiting* | Ref |  |
| *Separated/divorced* | 1.54 | Low |
| *Widowed* | 1.32 | Low |
| *Single* | 3.87 | Low to moderate |
| **Mean VIF** | **2.35** | **Low overall** |

| **Table S16. Health-related factors** | | |
| --- | --- | --- |
|  | *VIF* | *Interpretation* |
| Smoking status |  |  |
| *Current smoker* | Ref |  |
| *Past smoker* | 1.54 | Low |
| *Non-smoker* | 1.04 | Low |
| BMI category |  |  |
| *Normal weight* | Ref |  |
| *Underweight* | 1.12 | Low |
| *Overweight* | 1.74 | Low |
| *Obesity* | 1.63 | Low |
| *Severe obesity* | 1.13 | Low |
| Age at diagnosis | 6.05 | Moderate to high |
| Female | 7.09 | Moderate to high |
| Ethnicity |  |  |
| *White* | Ref |  |
| *Mixed* | 1.07 | Low |
| *South Asian* | 1.08 | Low |
| *Black Caribbean* | 1.22 | Low |
| *Black African* | 1.40 | Low |
| *Black Other* | 1.39 | Low |
| *Other* | 1.24 | Low |
| IMD quintile |  |  |
| *1 (most deprived)* | Ref |  |
| *2* | 1.89 | Low |
| *3* | 2.01 | Low |
| *4* | 2.08 | Low |
| *5* | 2.01 | Low |
| **Mean VIF** | **2.04** | **Low overall** |

| **Table S17.** Clinical features | | |
| --- | --- | --- |
|  | *VIF* | *Interpretation* |
| SMI diagnosis |  |  |
| *Schizophrenia* | Reference |  |
| *Bipolar disorder* | 8.00 | Moderate to high |
| Mental health comorbidities |  |  |
| *F0* | 1.23 | Low |
| *F1* | 1.44 | Low |
| *F3 (excl. F30, F31)* | 1.29 | Low |
| *F4* | 1.19 | Low |
| *F5* | 1.05 | Low |
| *F6* | 1.19 | Low |
| *F7* | 1.10 | Low |
| *F8* | 1.10 | Low |
| ACSC admissions | 1.13 | Low |
| HoNOS substance use | 3.86 | Low to moderate |
| HoNOS physical illness | 4.91 | Low to moderate |
| HoNOS daily living | 4.60 | Low to moderate |
| Age at diagnosis | 9.07 | Moderate to high |
| Female | 9.06 | Moderate to high |
| Ethnicity |  |  |
| *White* | Reference |  |
| *Mixed* | 1.07 | Low |
| *South Asian* | 1.07 | Low |
| *Black Caribbean* | 1.22 | Low |
| *Black African* | 1.35 | Low |
| *Black Other* | 1.33 | Low |
| *Other* | 1.24 | Low |
| IMD quintile |  |  |
| *1 (most deprived)* | Reference |  |
| *2* | 2.03 | Low |
| *3* | 2.15 | Low |
| *4* | 2.21 | Low |
| *5* | 2.12 | Low |
| **Mean VIF** | **2.64** | Low overall |

| **Table S18.** Treatment and service use patterns | | |
| --- | --- | --- |
|  | *VIF* | *Interpretation* |
| Mental health service use |  |  |
| *Number of psychiatric inpatient admissions* | 4.58 | Low to moderate |
| *Psychiatric inpatient bed days* | 2.26 | Low |
| *HTT events* | 1.88 | Low |
| *CMHT events* | 2.28 | Low |
| *MHA ever* | 2.58 | Low |
| *Emergency events* | 1.65 | Low |
| Clozapine prescribed | 1.38 | Low |
| Number of antipsychotic types prescribed |  |  |
| *0* | Reference |  |
| *1* | 2.46 | Low |
| *2* | 1.98 | Low |
| *3* | 1.72 | Low |
| *4+* | 2.89 | Low |
| Age at diagnosis | 6.05 | Moderate to high |
| Female | 7.03 | Moderate to high |
| Ethnicity |  |  |
| *White* | Reference |  |
| *Mixed* | 1.06 | Low |
| *South Asian* | 1.07 | Low |
| *Black Caribbean* | 1.18 | Low |
| *Black African* | 1.34 | Low |
| *Black Other* | 1.32 | Low |
| *Other* | 1.24 | Low |
| IMD quintile |  |  |
| *1 (most deprived)* | Reference |  |
| *2* | 1.85 | Low |
| *3* | 1.91 | Low |
| *4* | 1.95 | Low |
| *5* | 1.91 | Low |
| **Mean VIF** | **2.33** | Low overall |

| **Table S19.** Final integrated model | | |
| --- | --- | --- |
|  | *VIF* | *Interpretation* |
| Age at diagnosis | 7.91 | Moderate to high |
| Ethnicity |  |  |
| *White* | Ref |  |
| *Mixed* | 1.08 | Low |
| *South Asian* | 1.07 | Low |
| *Black Caribbean* | 1.25 | Low |
| *Black African* | 1.41 | Low |
| *Black Other* | 1.45 | Low |
| *Other* | 1.23 | Low |
| IMD decile |  |  |
| *1 (most deprived)* | Ref |  |
| *2* | 2.54 | Low to moderate |
| *3* | 1.64 | Low |
| *4* | 1.22 | Low |
| *5* | 1.11 | Low |
| Marital status |  |  |
| *Married/cohabiting* | Ref |  |
| *Separated/divorced* | 1.62 | Low |
| *Widowed* | 1.34 | Low |
| *Single* | 4.91 | Low to moderate |
| BMI category |  |  |
| *Normal weight* | Ref |  |
| *Underweight* | 1.12 | Low |
| *Overweight* | 1.79 | Low |
| *Obesity* | 1.70 | Low |
| *Severe obesity* | 1.15 | Low |
| Mental health comorbidities |  |  |
| *F0* | 1.24 | Low |
| *F1* | 1.51 | Low |
| *F7* | 1.05 | Low |
| Clozapine prescription | 1.31 | Low |
| Mental health service utilisation |  |  |
| *Number of acute episodes* | 3.15 | Low to moderate |
| *CMHT events* | 2.53 | Low |
| *Emergency* | 1.74 | Low |
| *MHA ever* | 3.58 | Low to moderate |
| ACSC admissions | 1.14 | Low |
| HoNOS substance use | 3.90 | Low to moderate |
| HoNOS physical illness | 4.89 | Low to moderate |
| HoNOS daily living | 4.75 | Low to moderate |
| **Mean VIF** | **2.21** | **Low overall** |

Died before infection-related hospitalisation

(N=1042)

Infection-related hospitalisation

(N=2542)

Reached end of follow-up (31^st^ December 2019)

(N=16,411)

**Figure S1.** Participant flow diagram. Total person-time at risk: 119,178.6 years

Final sample: N=19,995 individuals included in analysis

53 removed as SMI diagnosis after study end date (31 December 2019) (included in error)

N=20,048 SLaM service users with valid SMI diagnosis recorded in study window (1 January 2007 to 31 December 2019), aged ≥16 years at the time of diagnosis, and with no prior dementia diagnosis.
